# Longitudinal Study of Cellular and Systemic Cytokine Signatures to Define the Dynamics of a Balanced Immune Environment During Disease Manifestation in Zika Virus–Infected Patients

**DOI:** 10.1093/infdis/jiy225

**Published:** 2018-04-16

**Authors:** Fok-Moon Lum, David C B Lye, Jeslin J L Tan, Bernett Lee, Po-Ying Chia, Tze-Kwang Chua, Siti N Amrun, Yiu-Wing Kam, Wearn-Xin Yee, Wei-Ping Ling, Vanessa W X Lim, Vincent J X Pang, Linda K Lee, Esther W H Mok, Chia-Yin Chong, Yee-Sin Leo, Lisa F P Ng

**Affiliations:** 1 Singapore Immunology Network, Agency for Science, Technology, and Research, Singapore; 2 Communicable Diseases Centre, Institute of Infectious Diseases and Epidemiology, Tan Tock Seng Hospital, Singapore; 3 Lee Kong Chian School of Medicine, Nanyang Technological University, Singapore; 4 Department of Medicine, Yong Loo Lin School of Medicine, Singapore; 5 KK Women’s and Children’s Hospital, Singapore; 6 Saw Swee Hock School of Public Health, National University of Singapore, Singapore; 7 Department of Biochemistry, Yong Loo Lin School of Medicine, National University of Singapore, Singapore; 8 National Institute of Health Research, Health Protection Research Unit in Emerging and Zoonotic Infections, Liverpool, United Kingdom; 9 Institute of Infection and Global Health, University of Liverpool, United Kingdom

**Keywords:** Zika virus, patient cohort, cytokines, immunophenotyping, viremia

## Abstract

**Background:**

Since its unexpected reemergence, Zika virus (ZIKV) has caused numerous outbreaks globally. This study characterized the host immune responses during ZIKV infection.

**Methods:**

Patient samples were collected longitudinally during the acute, convalescence and recovery phases of ZIKV infection over 6 months during the Singapore outbreak in late 2016. Plasma immune mediators were profiled via multiplex microbead assay, while changes in blood cell numbers were determined with immunophenotyping.

**Results:**

Data showed the involvement of various immune mediators during acute ZIKV infection accompanied by a general reduction in blood cell numbers for all immune subsets except CD14^+^ monocytes. Importantly, viremic patients experiencing moderate symptoms had significantly higher quantities of interferon γ–induced protein 10, monocyte chemotactic protein 1, interleukin 1 receptor antagonist, interleukin 8, and placental growth factor 1, accompanied by reduced numbers of peripheral CD8^+^ T cells, CD4^+^ T cells, and double-negative T cells. Levels of T-cell associated mediators, including interferon γ–induced protein 10, interferon γ, and interleukin 10, were high in recovery phases of ZIKV infection, suggesting a functional role for T cells. The identification of different markers at specific disease phases emphasizes the dynamics of a balanced cytokine environment in disease progression.

**Conclusions:**

This is the first comprehensive study that highlights specific cellular changes and immune signatures during ZIKV disease progression, and it provides valuable insights into ZIKV immunopathogenesis.

## Teaser

Understanding the host immune response during Zika virus (ZIKV) infection will provide valuable insights into ZIKV immunopathogenesis. In this study, characterization of cellular changes and immune mediators of ZIKV-infected patients was performed, allowing for the identification of key infection-associated markers.

Zika virus (ZIKV) was an obscure flavivirus until it reemerged in 2015, with accompanying unexpected severe complications [1–3]. ZIKV is an arbovirus transmitted via the bite of infected *Aedes* mosquitoes, although non–vector‐borne transmissions such as sexual, maternal-fetal, and blood transfusion transmissions have been reported [4–7]. Typically, ZIKV infection is rarely life threatening, manifesting as a transient fever accompanied by headache, arthralgia, conjunctivitis, fatigue, and rash, with many patients being asymptomatic [3, 8]. ZIKV has been associated with neurological complications, such as Guillain-Barré syndrome in adults and congenital fetal growth abnormalities in newborns [1, 3, 9, 10].

While efforts have been made to study ZIKV immunopathogenesis, there is still a gap in knowledge of how patients respond immunologically to the infection. Previous investigation into ex vivo CD14^+^ monocytes [11] and monocyte-derived macrophages showed significant differences in messenger RNA transcript abundance after ZIKV infection [11–13]. However, the overall regulation of other immune subsets during disease progression and their associations with soluble immune mediators remain to be elucidated.

In this study, the focus is on characterizing immune markers in a cohort of ZIKV-infected patients recruited from the first ZIKV outbreak in Singapore, in 2016 [14, 15]. While none of the patients had severe disease, some presented with moderate symptoms. In-depth investigation was performed with longitudinal profiling of immune mediators present in the patients’ plasma collected during the acute to recovery phases of infection. This was further complemented with extensive immunophenotyping of whole-blood specimens collected from patients. This is the first comprehensive study that combines cellular changes with specific immune signatures during ZIKV infection, and it provides a better understanding on the pathobiology of the virus during infection. In addition, specific immune signatures allow for the prospect of identifying key markers associated with different disease manifestations.

## METHODS

### Standard Protocol Approvals, Registrations, and Patient Consent

Written informed consent was obtained from all participants in accordance with the Declaration of Helsinki for Human Research. Study protocols were approved by the SingHealth Centralized Institutional Review Board (reference 2016/2219) and the National Healthcare Group Domain Specific Review Board (reference 2015/00528). Collection of blood samples from healthy donors was done with written consent in accordance with guidelines from the Health Sciences Authority of Singapore (study approval number National University Singapore Institutional Review Board approval 10-250).

### Patients and Sample Collection

The first case of ZIKV infection was reported on 27 August 2016 [15], and detection was confirmed by ZIKV-specific quantitative reverse transcription polymerase chain reaction (qRT-PCR) analysis [16]. Thereafter, suspected ZIKV cases were referred/admitted to the Communicable Disease Centre at Tan Tock Seng Hospital for ZIKV testing. Hematological and biochemistry laboratory tests were performed in parallel with ZIKV-specific RT-PCR analysis [16] upon admission. ZIKV infection was confirmed by a positive result of ZIKV-specific RT-PCR analysis of whole-blood specimens. Subsequently, whole-blood specimens were obtained at 5 collection time points: (1) the acute phase (2–7 days after illness onset), (2) the early convalescent phase (10–14 days), (3) the late convalescent phase (25–35 days), (4) the early recovery phase (2–4 months), and (5) the late recovery phase (5–6 months). Whole-blood specimens were collected in ethylenediaminetetraacetic acid–coated Vacutainer tubes (Becton Dickinson) after peripheral venipuncture. Two milliliters of whole blood was first aliquoted for blood count analysis, whole-blood staining, and viral load quantification, and the remaining sample was centrifuged at 240*g* for 10 minutes to collect plasma for storage at −80°C. Samples from healthy donors were included and prescreened for the presence of ZIKV viral RNA [16] and ZIKV-specific antibodies [17]. All healthy donors were nonfebrile and had no signs of acute illness during recruitment.

### Blood Count

Complete blood count was performed using the Ac·T diff hematology analyzer (Beckman Coulter) according to the manufacturer’s instructions. Beckman Coulter 4C Plus Tri-Pack Cell Controls (Beckman Coulter) were conducted to confirm instrument accuracy and precision performance.

### Whole-Blood Staining and Flow Cytometry

Staining was performed on 100-μL whole-blood specimens from 44 ZIKV-infected patients and 14 healthy donors. Antibodies were used to identify CD45^+^ leukocytes (mouse anti-human CD45; Biolegend), CD16^+^ neutrophils (mouse anti-human CD16; Biolegend), CD14^+^ monocytes (mouse anti-human CD14; BD Biosciences), CD3^+^ T cells (mouse anti-human CD3; BD Biosciences), CD4^+^ helper T cells (mouse anti-human CD4; eBioscience), CD8^+^ cytotoxic T cells (mouse anti-human CD8; BD Biosciences), CD56^+^ natural killer (NK) cells (mouse anti-human CD56; Miltenyi Biotec), and CD19^+^ B cells (mouse anti-human CD19; eBioscience). Subsequently, cell fixation and red blood cells lysis were performed with 1X FACS lysing solution (BD Biosciences). Permeabilization was achieved with 1X FACS permeabilization solution 2 (BD Biosciences), after which cells were stained with ZIKV NS3 protein–specific rabbit polyclonal antibody [14]. Stained cells were counterstained with a fluorophore-tagged secondary goat anti-rabbit IgG (H+L) antibody (Invitrogen), before acquisition with LSR Fortessa analyzers, LSRII 4 lasers, and LSRII 5 lasers (BD Biosciences). Since samples were transported on ice within 3 hours to the laboratory at the Singapore Immunology Network, live/dead staining was not implemented because freshly collected whole-blood specimens typically contain very few dead cells [14]. Numbers of peripheral blood cell immune subsets were subsequently obtained with the following formula: [percentage of a specific immune subset, obtained from immunophenotyping] × [total leukocyte count, obtained from complete blood count] = the number of cells in a specific immune subset.

### ZIKV Load Quantification

RNA samples were extracted from 140-μL whole-blood samples, using the QIAamp Viral RNA Mini Kit (Qiagen) according to the manufacturer’s protocols. ZIKV quantification was performed by 1-step TaqMan real-time RT-PCR analysis (QuantiTect Probe RT-PCR Kit; Qiagen) as described previously [18, 19].

### Multiplex Microbead Immunoassay for Cytokine Quantification

Cytokine and chemokine levels in ZIKV-infected patients’ plasma were measured simultaneously, using a multiplex microbead-based immunoassay (ProcartaPlex Human Cytokine/Chemokine/Growth Factor Panel 1; Thermo Scientific) as described previously [18]. Preparation of plasma samples and reagents, as well as immunoassay procedures, were performed according to manufacturers’ instructions.

### Data Processing and Statistical Analysis

Luminex assay–determined concentrations obtained via Bio-plex Manager software (using 5-parameter logistic curve fitting) were normalized using median centering to remove potential plate effects. Each analyte was normalized separately, such that the median concentration for each plate was first determined and then the global median concentration from all plates was computed. A scaling factor was then computed for each plate, which adjusted the median concentration for the plate to that of the global median concentration. The final adjusted concentrations were then logarithmically transformed to assume a normal distribution before further data analysis and visualization.

Two-way analysis of variance (ANOVA) with the post hoc Tukey test was used to detect differences between the various sample groups and collections. ANOVA results were corrected for multiple testing, using the method of Benjamini and Hochberg. Nonparametric testing was done for cell percentages and viral load data, using the Mann-Whitney test or the Kruskal-Wallis test with Dunn multiple comparison tests. Data processing was done in the R statistical language (version 3.3.1). The relationship between analytes at different collections was also determined using Pearson correlation analysis. All statistical analyses were performed using R version 3.3.1 or Prism 7.0 (GraphPad software).

Hierarchical clustering and heat map visualization were done using TM4-MeV [20]. In the heat map presentation, the average concentration was computed for each measured analyte in its respective group, and the average values were then scaled between 0 and 1 for visualization [18].

Receiver operating characteristic (ROC) curve analysis for analytes that were differentially expressed between ZIKV-infected patients and healthy controls was performed, and the areas under the curve (AUCs) were calculated. Analytes with AUCs of >0.65 and a *P* value of <.05 are considered to be potential markers of infection.

A multivariate model was built using patient demographic characteristics, immune mediator profiles, and immunophenotyping data. The analysis was done using a logistic regression fit optimized for analysis by the Akaike information criterion (AIC) in a step-forward fashion. The number of steps was limited to a floor value (calculated as the number of samples/10), to ensure that there were sufficient data for fitting. Leave-one-out cross-validation was then used to assess the predictive quality of the model. Biological processes were predicted from differentially expressed mediators with Ingenuity Pathway Analysis (IPA; Qiagen). Interaction networks of selected immune mediators were predicted with STRING (version 10.5; available at: https://string-db.org/).

## RESULTS

### Demographic and Clinical Characteristics of ZIKV-Infected Patients

A total of 55 patients were recruited for the study, based on confirmatory results of ZIKV-specific PCR analysis upon hospitalization, and were subsequently recruited into the study. The majority of the patients returned for their follow-up consultations during the acute phase (55 patients), the early convalescent phase (34), the late convalescent phase (36), the early recovery phase (35), and the late recovery phase (32). Patient age ranged from 16 to 65 years (mean, 38 years; median, 35.0 years; interquartile range [IQR], 28–47 years), with the highest incidence of ZIKV infection occurring among patients aged 18–34 years (26 [47.3%]). Among the 55 patients, 26 (47.3%) still had ZIKV RNA detected by ZIKV-specific RT-PCR analysis (median, 16.8 viral copies/uL; IQR, 10.0–30.6 viral copies/uL) during the acute phase (Table 1). No neurological complications were observed in the patients during the acute phase of infection.

**Table 1.** Demographic and Clinical Characteristics of 55 Patients Infected With Zika Virus (ZIKV)

Variable	Value
Age, y	
16–34	26 (47.3)
35–54	22 (40.0)
≥55	7 (12.7)
Overall	35 (28–47)
Sex	
Female	24 (43.6)
Male	31 (56.4)
Ethnicity	
Chinese	44 (80.0)
Indian	3 (5.45)
Malay	4 (7.27)
Others	4 (7.27)
ZIKV viremia status^a^	
Viremic	26 (47.3)
Nonviremic	29 (52.7)
Symptoms^b^	
Fever	39 (70.9)
Rash	50 (90.9)
Myalgia	29 (52.7)
Arthralgia	24 (43.6)
Headache	19 (34.5)
Conjunctivitis	19 (34.5)
Sore throat	9 (16.4)
Cough	8 (14.5)
ZIKV RNA level, viral copies/uL ^a^	16.8 (10.0–30.6)
Hospitalization duration, d	2 (2–3)
Laboratory test results	
Na level, mmol/L	138 (136–140)
K level, mmol/L	3.6 (3.4–3.7)
Urea level, mmol/L	3.3 (2.8–3.7)
Creatinine level, mmol/L	73 (57–85)
AST level, U/L	26 (20–30)
ALT level, U/L	20 (16–31)
Blood test results	
Hemoglobin level, g/dL	14.1 (13.4–15.2)
Hematocrit, %	42.8 (40.3–46.0)
Platelet count, ×10^3^ platelets/µL	202.5 (176.8–238.5)
Bilirubin level, µmol/L	11 (9–14)
Albumin level, g/L	39 (37–43)

Data are no. (%) of patients or median value (interquartile range).

Abbreviations: ALT, alanine aminotransferase; AST, aspartate aminotransferase.

^a^The ZIKV RNA load was determined during acute phase of infection and is expressed as viral copies/uL of extracted viral RNA.

^b^Among the symptoms, rash (in 50 patients [90.9%]) and fever (in 39 [70.9%]) were the most prevalent during hospital admission, followed by myalgia (in 29 [52.7%]), arthralgia (in 24 [43.6%]), headache (in 19 [34.5%]), cough (in 8 [14.5%]), and sore throat (in 9 [16.4%]). Conjunctivitis (of ocular conjunctiva) was observed in 19 patients (34.5%). Symptoms were defined as moderate if a patient presented with >4 of 8 symptoms.

### ZIKV Infection Triggers High Levels of Proinflammatory Mediators

**Figure 1.**
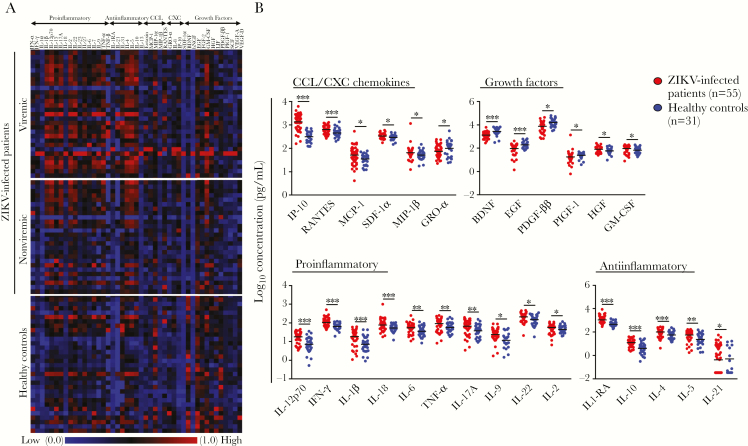
Profiling of immune mediators in plasma from patients with acute Zika virus (ZIKV) infection. A microbead assay was performed to quantify the levels of immune mediators in plasma specimens obtained from 55 patients during the acute phase of infection. *A*, Levels of 45 immune mediators were analyzed and presented in a heat map of normalized scores (0–1). *B*, Box-and-whisker plots of levels of 27 mediators that were statistically significantly different between ZIKV-infected patients and 31 healthy controls. Concentrations of immune mediators are expressed on a log_10_ scale. *P < .05, **P < .01, and ***P < .001 by *t* tests conducted on the logarithmically transformed concentrations, with correction for multiple testing by the Benjamini-Hochberg method. BDNF, brain-derived neurotrophic factor; bNGF, β nerve growth factor; EGF, epidermal growth factor; FGF-2, fibroblast growth factor 2; GM-CSF, granulocyte-macrophage colony-stimulating factor; GRO-α, growth-regulated protein α; HGF, hepatocyte growth factor; IFN-α, interferon α; IFN-γ, interferon γ; IL-1RA, interleukin 1 receptor antagonist; IL-1α, interleukin 1α; IL-1β, interleukin 1β; IL-2, interleukin 2; IL-4, interleukin 4; IL-5, interleukin 5; IL-6, interleukin 6; IL-7, interleukin 7; IL-8, interleukin 8; IL-9, interleukin 9; IL-10, interleukin 10; IL-12p70, interleukin 12p70; IL-13, interleukin 13; IL-15, interleukin 15; IL-17A, interleukin 17A; IL-18, interleukin 18; IL-21, interleukin 21; IL-22, interleukin 22; IL-23, interleukin 23; IL-27, interleukin 27; IL-31, interleukin 31; IP-10, interferon γ–induced protein 10; LIF, leukemia inhibitory factor; MCP-1, monocyte chemotactic protein 1; MIP-1α, macrophage inflammatory protein 1α; MIP-1β, macrophage inflammatory protein 1β; PDGF-ββ, platelet-derived growth factor ββ; PIGF-1, placental growth factor 1; RANTES, regulated on activation normal T-cell expressed and secreted; SCF, stem cell factor; SDF-1α, stromal cell–derived factor 1α; TNF-α, tumor necrosis factor α; TNF-β, tumor necrosis factor β; VEGF-A, vascular endothelial growth factor A; VEGF-D, vascular endothelial growth factor D.

To define the acute host immune markers during ZIKV infection, levels of immune mediators present in patients’ plasma obtained during the acute phase were quantified by a 45-plex microbead assay (Figure 1A). It was observed that patients with acute ZIKV infection had significantly higher levels of CCL/CXC chemokines (ie, interferon γ [IFN-γ]–induced protein 10 [IP-10], regulated on activation normal T-cell expressed and secreted [RANTES], monocyte chemoattractant protein 1 [MCP-1], stromal cell–derived factor 1α [SDF-1α], macrophage inflammatory protein 1β [MIP-1β], and growth-regulated oncogene α [GRO-α]), growth factors (ie, brain-derived neurotrophic factor [BDNF], epidermal growth factor [EGF], platelet-derived growth factor ββ [PDGF-ββ], placenta growth factor 1 [PIGF-1], hepatocyte growth factor [HGF], and granulocyte-macrophage colony-stimulating factor [GM-CSF]), proinflammatory cytokines (ie, interleukin 12p70 [IL-12p70], IFN-γ, interleukin 1β [IL-1β], interleukin 18 [IL-18], interleukin 6 [IL-6], tumor necrosis factor α [TNF-α], interleukin 17A [IL-17A], interleukin 9 [IL-9], interleukin 22 [IL-22], and interleukin 2 [IL-2]), and antiinflammatory cytokines (ie, IL-1 receptor antagonist [IL-1RA], interleukin 10 [IL-10], interleukin 4 [IL-4], interleukin 5 [IL-5], and interleukin 21 [IL-21]) than healthy controls (Figure 1B). Further categorization of ZIKV-infected patients on the basis of viremic status (Table 1) revealed differences between viremic and nonviremic patients (Figure 1A and Supplementary Table 1). Collectively, these data highlight the presence of unique cytokine profiles among ZIKV-infected patients.

### ZIKV Infection Reduces the Peripheral Blood Cell Count

**Figure 2.**
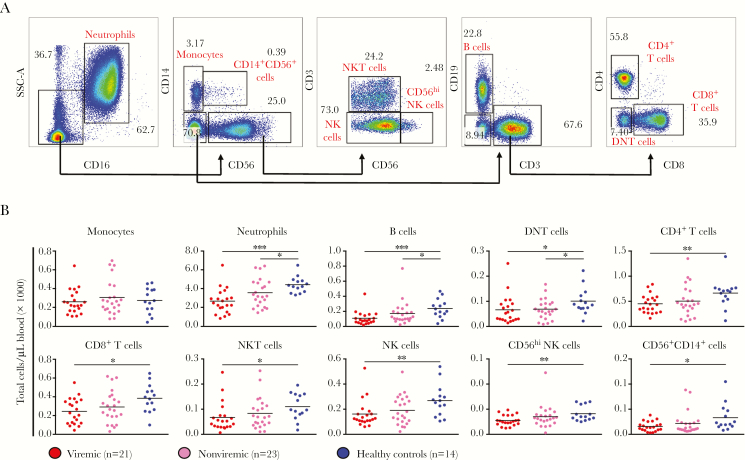
Blood immunophenotyping of patients with acute Zika virus (ZIKV) infection. Percentages of specific immune subsets were assessed via immunophenotyping of whole-blood specimens. *A*, Gating strategy of immunophenotyping. Briefly, immune cells were identified with a combination of CD45, CD14, CD3, CD4, CD8, CD19, CD16, and CD56 antibodies. Data in the plots shown were obtained from a representative donor. *B*, The cellular numbers of each identified immune subset in the peripheral blood were subsequently determined by the following formula: [percentages of specific immune subset] × [total leukocyte numbers] = cellular numbers of specific immune subset. Total leukocyte numbers were obtained with a hematology analyzer. Data were obtained from 21 viremic and 23 nonviremic patients. Values obtained from 14 healthy controls are plotted alongside for comparison. *P < .05, **P < .01, and ***P < .001 by 2-tailed nonparametric Mann-Whitney tests. DNT, double-negative T; NK, natural killer; NKT, natural killer T; SSC, side scatter.

Blood cell count and subset profiles are important criteria for determining the diagnosis and prognosis of viral infections [21]. Changes in the number of neutrophils (CD45^+^CD16^+^), monocytes (CD14^+^), B cells (CD19^+^), T cells (CD3^+^CD4^+^, CD3^+^CD8^+^), double-negative (CD3^+^CD4^-^CD8^-^) T (DNT) cells, NK cells (CD56^+^ and CD56^hi^) NK T cells (CD56^+^CD3^+^), and CD14^+^CD56^+^ cells during acute ZIKV infection were profiled (Figure 2A).

It was observed that, during acute ZIKV infection, the total number of blood leukocytes in the patients was significantly lower than in healthy controls, owing to the overall reduction of all immune populations except monocytes (Figure 2B). It was observed that ZIKV-infected patients generally had decreased numbers of B cells, DNT cells, and neutrophils. The presence of detectable viremia affected the numbers of other immune subsets (Figure 2B), while in nonviremic patients, an intermediate phenotype (ie, a nonsignificant drop in cell number) was observed. Notably, although ZIKV-infected patients had leukopenia, the number of cells in each immune subset was still within the normal range [22].

### Unique Cellular and Immune Mediator Signatures Associated With Disease Symptoms

**Figure 3.**
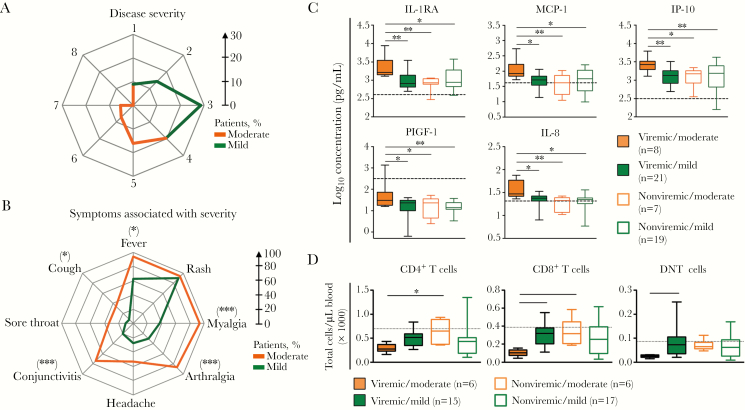
Specific immune response in Zika virus (ZIKV)–infected patients categorized by symptom manifestations. *A* and *B*, Radar charts showing the percentages of patients experiencing either mild or moderate symptoms, based on their clinical scores (*A*), and the differences in the prevalence of symptom manifestations between patients in the 2 groups (*B*). Clinical scores reflect the numbers of symptoms experienced during admission. *P < .05, **P < .01, and ***P < .001 by 2-sided Fisher exact tests for comparison of the prevalence of symptoms between groups. *C* and *D*, Box-and-whisker plots of levels of specific cytokines (*C*) and immune subsets (*D*) that were significantly different between patients in the viremic/moderate group and patients from the other 3 groups (ie, viremic/mild, nonviremic/moderate, and nonviremic/mild). *P < .05 and **P < .01 by 1-way analysis of variance, with correction for multiple testing by the Benjamini-Hochberg method with post hoc Tukey test (for cytokines), or by the Kruskal-Wallis test, with the Dunn multiple comparison test (for immune subsets). DNT, double-negative T; IL-1RA, interleukin 1 receptor antagonist; IL-8, interleukin 8; IP-10, interferon γ–induced protein 10; MCP-1, monocyte chemotactic protein 1; PIGF-1, placental growth factor 1.

Because the ZIKV epidemic was anticipated, a study was planned with a standardized care path to collect demographic data, comorbidity data, hospitalization details, travel history and sick contact, pregnancy and vaccination status, signs and symptoms, and laboratory data on complete blood count; renal and liver function test results; diagnostic test results for ZIKV, dengue virus, and chikungunya virus; and clinical outcomes (ie, full recovery, sequelae, and death). In this study, symptoms were defined as moderate if a patient presented with >4 of 8 symptoms presented in Table 1. Patients who did not fulfill this criterion were classified as having mild symptoms (Figure 3A). It was observed that patients with moderate ZIKV disease experienced higher incidences of myalgia, arthralgia, conjunctivitis, and cough (Figure 3B). Rash was present in >90% of patients from both groups (Figure 3B).

**Figure 4.**
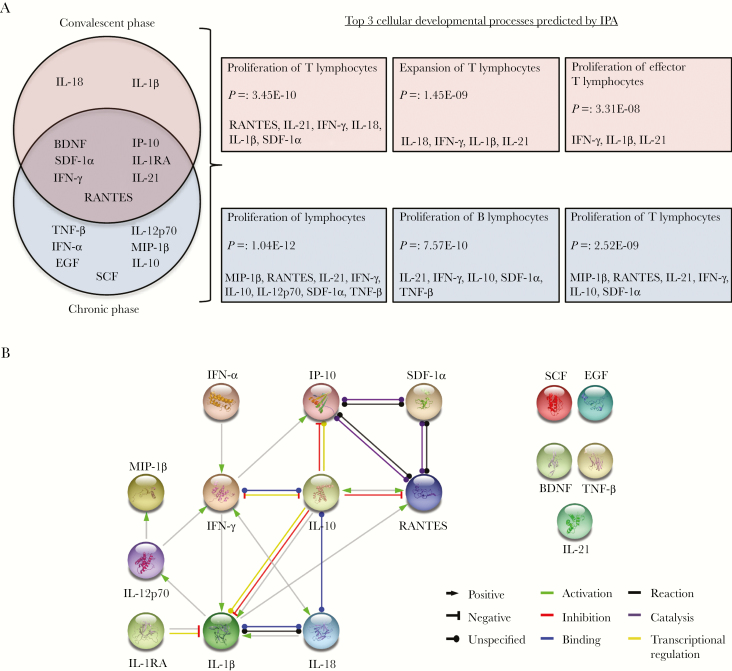
Immune response in Zika virus (ZIKV)–infected patients after the acute phase of infection. Immune mediators during the convalescent and recovery phases were assessed via a microbead-based assay. *A*, Venn diagram displaying the mediators that are differentially expressed in ZIKV-infected patients as compared to healthy controls during the convalescent and recovery phases of infection. Shown are the top 3 cellular developmental processes and associated mediators, as predicted with Ingenuity Pathway Analysis (IPA). *B*, Interactive relationships between the immune mediators were determined by STRING analysis, with a confidence threshold of .85. BDNF, brain-derived neurotrophic factor; EGF, epidermal growth factor; IFN-α, interferon α; IFN-γ, interferon γ; IL-1RA, interleukin 1 receptor antagonist; IL-1β, interleukin 1β; IL-10, interleukin 10; IL-12p70, interleukin 12p70; IL-18, interleukin 18; IL-21, interleukin 21; IP-10, interferon γ–induced protein 10; MIP-1β, macrophage inflammatory protein 1β; RANTES, regulated on activation normal T-cell expressed and secreted; SCF, stem cell factor; SDF-1α, stromal cell–derived factor 1α; TNF-β, tumor necrosis factor β.

To further identify specific unique immune profiles, ZIKV-infected patients were categorized on the basis of both their viremic status and symptom manifestation, as follows: (1) nonviremic with mild symptoms, (2) nonviremic with moderate symptoms, (3) viremic with mild symptoms, and (4) viremic with moderate symptoms. Viremic patients with moderate symptoms had significantly higher levels of IL-1RA, MCP-1, IP-10, and PIGF-1, compared with other patients. These patients also had increased levels of IL-8 (Figure 3C and Supplementary Table 2). Levels of these immune mediators were relatively similar among the other 3 groups.

In terms of total blood profiling, viremic patients experiencing moderate symptoms of ZIKV infection had reduced numbers of DNT cells, CD4^+^ T cells, and CD8^+^ T cells (Figure 3D). To further understand the link between the host immune response and ZIKV disease progression, multivariate prediction on ZIKV viremic status and disease outcome was conducted using data from both the microbead assay and immunophenotyping. The analysis predicted that HGF could influence disease outcome (Supplementary Table 3). On the other hand, the percentages of monocytes and CD56^hi^ NK cells were predicted to influence symptom severity, whereas neutrophils were predicted to influence viremia (Supplementary Table 3).

### Levels of T-Cell–Associated Cytokines and Chemokines Remain High After the Acute Phase of ZIKV Infection

Blood immunophenotyping findings and immune markers were further profiled longitudinally during the acute, early and late convalescent, and early and late recovery phases among ZIKV-infected patients (Figure 4A). Levels of 14 immune mediators were found to be significantly higher than those in healthy controls during acute ZIKV infection. Of these, 13 could be potential markers for acute ZIKV infection, as calculated by ROC analyses that evaluated their diagnostic values (Supplementary Table 4). Uniquely, there exists a group of immune mediators that remain differentially expressed from the acute phase through the postacute phases of ZIKV infection (Table 2). Although levels in the majority (ie, in IP-10, IL-10, IL-1RA, IL-12p70, IL-1β, RANTES, and IFN-γ) peaked during the acute phase, levels peaked in later phases for some (ie, IL-18 [convalescent phase] and SDF-1α, IL-21, and MIP-1β [recovery phase]). These immune mediators could potentially be useful markers of these phases, based on ROC analyses (Table 2). Interestingly, levels of IL-21 were significantly lower during the acute phase and peaked only during the recovery phase, making it a potential marker for the recovery phase (Table 2).

**Table 2.** Analysis of Immune Mediator Levels Among Healthy Controls and Zika Virus (ZIKV)–Infected Patients During the Postacute Phases of ZIKV Infection

Phase, Immune Mediator	Level, Log_10_ pg/mL, Mean		Adjusted *P*^a^	ROC Analysis, AUC (95% CI)		*P* ^b^
	Healthy Controls	ZIKV-Infected Patients				
Early convalescent						
IP-10	2.5866	2.8702	<.0001	0.8833 (.8037–.9629)		<.0001
IL-1RA	2.6719	2.8447	.0025	0.7571	(.6362–.8780)	.0004
IL-21	0.2904	-0.6542	.0155	0.5705	(.4080–.7330)	.4326
IL-18	1.7089	1.9178	.0182	0.6917	(.5621–.8212)	.0080
SDF-1α	2.4688	2.5328	.0357	0.6667	(.5309–.8024)	.0220
IFN-γ	1.8324	1.9940	.0427	0.7097	(.5809–.8384)	.0037
Late convalescent						
IFN-γ	1.8324	2.0241	.0027	0.7608	(.6464–.8751)	.0003
IL-1RA	2.6719	2.8271	.0027	0.7401	(.6204–.8599)	.0008
IL-18	1.7089	1.9431	.0027	0.7401	(.6204–.8599)	.0008
RANTES	2.6539	2.7768	.0063	0.6254	(.4871–.7638)	.0783
BDNF	3.4277	3.2368	.0118	0.7384	(.6153–.8614)	.0008
IL-1β	0.9149	1.1365	.0439	0.5021	(.3438–.6604)	.9791
Early recovery						
IP-10	2.5866	2.7809	<.0001	0.8700	(.7831–.9570)	<.0001
TNF-β	-0.3277	-1.1561	.0003	0.7300	(.6033–.8566)	.0013
IL-1RA	2.6719	2.9355	.0008	0.7862	(.6750–.8974)	<.0001
IL-21	0.2904	0.9240	.0143	0.7972	(.6421–.9523)	.0019
IFN-γ	1.8324	2.0087	.0182	0.6931	(.5654–.8208)	.0071
RANTES	2.6539	2.7696	.0182	0.6986	(.5693–.8280)	.0056
IFN-α	-0.2438	-0.6617	.0182	0.6645	(.5282–.8008)	.0273
SDF-1α	2.4688	2.5734	.0236	0.7088	(.5838–.8337)	.0036
IL-12p70	0.9016	1.0991	.0236	0.6793	(.5493–.8092)	.0125
MIP-1β	1.7129	1.8926	.0236	0.6857	(.5578–.8136)	.0096
BDNF	3.4277	3.2787	.0272	0.7318	(.6064–.8572)	.0012
IL-10	0.6270	0.8388	.0441	0.6537	(.5173–.7901)	.0334
Late recovery						
TNF-β	-0.3277	-1.1325	.0022	0.7211	(.5900–.8523)	.0028
IFN-α	-0.2438	-0.7634	.0072	0.7071	(.5737–.8404)	.0051
** **IP-10	2.5866	2.7448	.0072	0.8216	(.7172–.9260)	.0001
EGF	2.3015	2.1273	.0159	0.6744	(.5415–.8073)	.0174
IL-1RA	2.6719	2.8726	.0159	0.747	(.6222–.8718)	.0008
BDNF	3.4277	3.2442	.0202	0.7419	(.6154–.8685)	.0010
SCF	0.9699	0.8801	.0241	0.6855	(.5540–.8170)	.0114

Abbreviations: AUC, area under the curve; BDNF, brain-derived neurotrophic factor; CI, confidence interval; EGF, epidermal growth factor; IFN-α, interferon α; IFN-γ, interferon γ; IP-10, interferon γ–induced protein 10; IL-1RA, interleukin 1 receptor antagonist; IL-1β, interleukin 1β; IL-10, interleukin 10; IL-12p70, interleukin 12p70; IL-18, interleukin 18; IL-21, interleukin 21; MIP-1β, macrophage inflammatory protein 1β; RANTES, regulated on activation normal T-cell expressed and secreted; ROC, receiver operating characteristic; SCF, stem cell factor; SDF-1α, stromal cell–derived factor 1α; TNF-α, tumor necrosis factor α; TNF-β, tumor necrosis factor β.

^a^By the Student *t* test, with correction for multiple testing, using the Benjamini-Hochberg method.

^b^Immune mediators with a *P* value < .05 are considered to exhibit a reasonable AUC, signifying their potential as a marker during the different phases of ZIKV infection.

To further understand the consequences of these differentially expressed immune mediators in ZIKV-infected patients, cellular developmental processes were predicted using IPA. It was observed that processes involving proliferation and expansion of T cells were important (Figure 4A). However, blood cell counts at both the convalescent and recovery phases did not indicate any significant differences between ZIKV-infected patients and healthy controls for all immune subsets.

## DISCUSSION

In this study, ZIKV-infected patients from the Singapore cohort had high levels of proinflammatory mediators, antiinflammatory mediators, CCL/CXC chemokines, and growth factors. While this robust immune response was expected, it is worthwhile to note that high levels of IFN-γ, IL-18, IL-10, IP-10, and TNF-α are consistent with our earlier report detailing the immune mediator profile from a Brazilian patient cohort [18]. Uniquely, high levels of GM-CSF, IL-2, IL-4, IL-5, IL-6 IL-9, IL-17A, IL-22, MCP-1, HGF, and TNF-α and low levels of PIGF-1 and PGDF-ββ were identified only in the acute phase, making them useful markers for acute ZIKV infection. As such, the prognostic and predictive values on the combinatorial assessment of these markers should be further understood to improve patient management.

The duration of detectable acute ZIKV viremia is relatively short [23]. With this in mind, ZIKV-infected patients were further grouped on the basis of both viremic status and symptom manifestations. Viremic patients with moderate disease exhibited high levels of IL-1RA, MCP-1, IP-10, PIGF-1, and IL-8. IL-8 could be a symptom severity marker only in such patients, indicating that subtle unique differences could be missed if detailed clinical and laboratory parameters are incomplete. However, in this study, it was observed that the window for detecting viremic patients with moderate symptoms is short, because levels of IL-1RA, IL-8, PIGF-1, MCP-1, and IP-10 were no longer significant after the acute phase (Supplementary Table 2). While the majority of the aforementioned immune mediators have been previously associated with dengue virus infection [24, 25], PIGF-1 could have an important role in ZIKV-induced congenital abnormalities, because placental PIGF-1 has roles in vasculogenesis and angiogenesis in placenta [26]. Coincidentally, levels of MCP-1, IP-10, IL-1RA, and IL-8 were higher in patients with severe dengue [24, 27], similar to what was observed in this cohort of ZIKV-infected patients.

Hematological changes have commonly been reported for other arboviral infections, such as those due to DENV and chikungunya virus, but not for ZIKV infection. In this cohort, it was observed that the patients experienced transient leukopenia and neutropenia, possibly due to transmigration of immune cells into the tissues during acute infection. The role of blood immune cells in ZIKV infection is understudied, but monocytes are a reported cellular target for ZIKV [11–14, 19]. Moreover, monocytes have been shown ex vivo to modulate the host immune response during ZIKV infection [13]. High levels of MCP-1 and GM-CSF detected in patients during the acute phase further confirm the role of monocytes during infection.

Lower numbers of DNT cells, CD4^+^ T cells, and CD8^+^ T cells were observed in viremic patients with moderate symptoms during the acute phase. This could be due to high levels of IP-10 in these patients, because IP-10 is responsible for recruitment of T cells to infection sites [28]. CD8^+^ T cells have been demonstrated to have a protective role during ZIKV infection in immunocompetent animals [29], and their cytotoxicity can be influenced by their responsiveness to IL-8 [30]. Likewise, higher IL-1RA levels in these ZIKV-infected patients could also signal regulated IL-1 signaling in the Th17 subset of the CD4^+^ T cells [31, 32]. On the other hand, little is known about the function of DNT cells in viral infections. DNT cells are commonly associated with autoimmune lymphoproliferative syndrome [33] and are known to share some characteristics with regulatory T cells and CD8^+^ T cells [33]. Their functional role in ZIKV infection should therefore not be neglected. The overall importance of T cells during acute ZIKV infection is further highlighted by the general presence of T-helper type 1 (Th1)–related (IL-2 and IFN-γ), Th2-related (IL-4), Th17-related (IL-17A), and Th9-related (IL-9) cytokines detected in this study, an observation that has been previously reported [34].

Functional prediction by IPA suggested active roles for T cells during the convalescent and recovery phases of ZIKV disease, while STRING analysis (performed with a high confidence threshold of 0.85) revealed key interactions between 11 of these immune mediators (Figure 4B). As expected, the predicted interactions are linked to T cells. IFN-γ, a known T-cell cytokine [35], was predicted to positively trigger the activity of IP-10 and IL-1β, which are mediators involved in T-cell recruitment and activation [28, 31, 36]. It was also predicted to interact with IL-10, an antiinflammatory cytokine [37], resulting in reciprocal inhibition. IFN-γ activity could in turn be triggered by IL-12p70 [38], IFN-α [39], and IL-18 [40]. IL-10, a regulator of immunity [41], was predicted to inhibit the activity of IP-10, RANTES [42], and IL-1β. Interestingly, the balance between IFN-γ and IL-10 has been reported to be critical in the pathogenesis of autoimmune diseases [43, 44]. Other interactions predicted by STRING analysis suggest binding and catalytic relationships between other T-cell chemokines, RANTES, SDF-1α and IP-10 [45]. Taken together, these findings emphasize the importance of a balanced cytokine environment involved in the T-cell response [46] during the postacute phases of ZIKV infection.

In this cohort, fever, conjunctivitis, arthralgia, and myalgia were the main symptoms in moderately symptomatic patients, in contrast to those reported for dengue virus infection [47]. The finding that viremic and moderately symptomatic patients had higher levels of IL-8, MCP-1, and IP-10 could possibly explain the higher incidence of conjunctivitis, through the recruitment of immune cells to the conjunctiva [48]. Moreover, these cytokines were reported to be associated with acute arthralgia and myalgia in chikungunya virus–infected patients [49, 50].

The marked differences in disease presentation between mildly and moderately symptomatic patients reported in this study are useful in developing a more robust definition of ZIKV infection. Importantly, the presence of viremia does not necessarily equate to more-severe disease. Likewise, symptomatic patients can be nonviremic, which suggests the interplay between symptoms, viremia, and host immune response in defining disease severity. Collectively, ZIKV infection stands out on its own apart from other flaviviruses, and the findings reported here will open up new research angles that can only increase knowledge about ZIKV immunopathogenesis.

## Supplementary Data

Supplementary materials are available at *The Journal of Infectious Diseases* online. Consisting of data provided by the authors to benefit the reader, the posted materials are not copyedited and are the sole responsibility of the authors, so questions or comments should be addressed to the corresponding author.

Supplementary Table 1

Supplementary Table 2

Supplementary Table 3

Supplementary Table 4

## Notes

***Acknowledgments.***  We thank Cheryl Y. P. Lee, Jonathan Cox, Yi-Hao Chan, Guillaume Carissimo, Farhana Abu Bakar, Nicholas Q. R. Kng, Kia-Joo Puan, and Nurhashikin Binte Yusof (Singapore Immunology Network [SIgN]), for their help in processing patient samples; Ivy Low, Seri Mustafah, and Anis Larbi (SIgN flow cytometry team) and the SIgN immunomonitoring group, for their assistance; Laurent Rénia, Teck-Hui Teo, and Kai-Er Eng, for critical discussion and proofreading of the manuscript; and all study participants and healthy volunteers, for their participation.

***Financial support.*** This work was supported by the Singapore Biomedical Research Council (BMRC; core research grants to the Singapore Immunology Network), the Singapore National Public Health Laboratory, the University of Tartu, and the BMRC and Singapore Agency for Science, Technology, and Research–led Zika Virus Consortium Fund (project 15/1/82/27/001).

***Potential conflicts of interest.*** All authors: No reported conflicts.
